# Comments on “Measuring What Matters in Parkinson's Disease Research and Dysphagia: The Need for Core Outcome Sets”

**DOI:** 10.1002/mds.70113

**Published:** 2025-11-01

**Authors:** Tao Xie, Lisa Bloom, Ellen McCracken

**Affiliations:** ^1^ Department of Neurology University of Chicago Medicine Chicago Illinois USA; ^2^ Speech and Swallow Section, Department of Otolaryngology, Head and Neck Surgery University of Chicago Medicine Chicago Illinois USA

**Keywords:** Dysphagia, Liquids, Early, Parkinson

We read with great interest the article by Walshe et al. entitled “Measuring what matters in Parkinson's disease research and dysphagia: the need for core outcome sets”.^1^ We strongly agree that core outcomes are needed in clinical research in dysphagia to improve the evidence base and decision making in patients with Parkinson's disease (PD), and that choking is an important item to be included in the core outcomes, as aspiration pneumonia is the primary cause of mortality in patients with PD.^1^, ^2^ While patient‐reported outcome measures provide useful guidance, it is important to also note that liquids pose a much higher and earlier‐presenting risk of aspiration than solid foods in patients with PD.^3^, ^4^ In a randomized, double‐blind, long‐term study, swallowing function was assessed with and without deep brain stimulation in PD patients treated with dopaminergic medications, and the depth (severity) and frequency of penetration and aspiration for liquids versus solids were compared by Penetration‐Aspiration Scale (PAS) during the videofluoroscopic swallow study.^3^ The severity and frequency of the incidence of penetration and aspiration were found to be much greater for liquids than solids, regardless of the conditions and time to assess.^3^ Aspiration of liquids was also found to occur earlier than that of solids even in patients with early‐stage PD.^4^ These findings suggest that liquids are more sensitive than solids for detecting early aspiration or deterioration in swallowing function. In progressive supranuclear palsy (PSP) too, dysphagia to liquids precedes that to solids,^5^ and is a major risk factor for mortality.^6^ In both clinical and research settings, asking a simple screening question such as “Do you cough when you drink water?” could help detect early aspiration. Emphasizing this question may alert physicians, researchers, patients, and families to dysphagia in its earliest stage, before more severe complications such as choking with solid foods or aspiration pneumonia develop. Objective or instrumental evaluations identify dysphagia with more specificity than subjective reports, suggesting that we should screen patients earlier in both research and clinical practice to facilitate earlier swallow studies. PD patients should have objective swallow function evaluated earlier given the much higher dysphagia prevalence found by objective evaluation compared with subjective report.^2^ Earlier referrals to speech pathology for evaluation may also improve swallowing outcomes.^7^ Including information on coughing with liquids (particularly thin liquids like water) with a probing question and subsequent swallow evaluation could also allow us to accumulate knowledge on early features of dysphagia and help achieve better clinical outcomes through early management in the future. Emphasizing this information may help not only patients with PD, but also PSP and possibly other neurodegenerative parkinsonian disorders too. We highly agree with characterizing the severity of oropharyngeal dysphagia with DIGEST and MBS ImP, together with PAS (which factors into the DIGEST rating) and pharyngeal residue ratings as planned in the Core Outcome Sets. We would also advocate using the ASPEKT (Analysis of Swallowing Physiology: Events, Kinematics, and Timing) to more precisely quantify the total pharyngeal residue as well.

## Author Roles

(1) Research Project: A. Conception, B. Organization, C. Execution; (2) Statistical Analysis: A. Design, B. Execution, C. Review and Critique; (3) Manuscript: A. Writing of the First Draft, B. Review and Critique.

T.X.: 1A, 1B, 1C, 2C, 3A, 3B.

L.B.: 1C, 2C, 3B.

E.M.: 1C, 2C, 3B.

## Financial Disclosures

Full Financial Disclosures of All Authors for the Preceding 12 Months: T.X.: Stock Ownership in medically related fields: None; Intellectual Property Rights: None; Consultancies: CVS Caremark; Expert Testimony: None; Advisory Boards: None; Employment: The University of Chicago Medicine; Partnerships: None; Inventions: None; Contracts: None; Honoraria: Parkinson's Foundation, Ono Pharmaceutical Co., Ltd, and CVS Caremark; Royalties: None; Patents: None; Grants: The Michael J. Fox Foundation for Parkinson's Research, American Parkinson's Disease Association, Cure PSP, and National Institutes of Health (NIH); Other: None. L.B.: Stock Ownership in medically related fields: None; Intellectual Property Rights: None; Consultancies: None; Expert Testimony: None; Advisory Boards: None; Employment: The University of Chicago Medicine; Partnerships: None; Inventions: None; Contracts: None; Honoraria: None; Royalties: None; Patents: None; Grants: NIH; Other: None. E.M.: Stock Ownership in medically related fields: None; Intellectual Property Rights: None; Consultancies: None; Expert Testimony: None; Advisory Boards: None; Employment: The University of Chicago Medicine; Partnerships: None; Inventions: None; Contracts: None; Honoraria: None; Royalties: None; Patents: None; Grants: NIH; Other: None.

## Data Availability

Data sharing not applicable – no new data generated.
